# Sea Urchin Extracellular Proteins Design a Complex Protein Corona on Titanium Dioxide Nanoparticle Surface Influencing Immune Cell Behavior

**DOI:** 10.3389/fimmu.2019.02261

**Published:** 2019-09-20

**Authors:** Andi Alijagic, Oldřich Benada, Olga Kofroňová, Diego Cigna, Annalisa Pinsino

**Affiliations:** ^1^Istituto per la Ricerca e l'Innovazione Biomedica (IRIB), Consiglio Nazionale delle Ricerche, Palermo, Italy; ^2^Institute of Microbiology of The Czech Academy of Sciences, Prague, Czechia

**Keywords:** echinoderm, biocorona, immune-adhesome, extracellular signaling, *in vitro-ex vivo* model, *proxy* to human

## Abstract

Extensive exploitation of titanium dioxide nanoparticles (TiO_2_NPs) augments rapid release into the marine environment. When in contact with the body fluids of marine invertebrates, TiO_2_NPs undergo a transformation and adhere various organic molecules that shape a complex protein corona prior to contacting cells and tissues. To elucidate the potential extracellular signals that may be involved in the particle recognition by immune cells of the sea urchin *Paracentrotus lividus*, we investigated the behavior of TiO_2_NPs in contact with extracellular proteins *in vitro*. Our findings indicate that TiO_2_NPs are able to interact with sea urchin proteins in both cell-free and cell-conditioned media. The two-dimensional proteome analysis of the protein corona bound to TiO_2_NP revealed that negatively charged proteins bound preferentially to the particles. The main constituents shaping the sea urchin cell-conditioned TiO_2_NP protein corona were proteins involved in cellular adhesion (*Pl*-toposome, *Pl*-galectin-8, *Pl*-nectin) and cytoskeletal organization (actin and tubulin). Immune cells (phagocytes) aggregated TiO_2_NPs on the outer cell surface and within well-organized vesicles without eliciting harmful effects on the biological activities of the cells. Cells showed an active metabolism, no oxidative stress or caspase activation. These results provide a new level of understanding of the extracellular proteins involved in the immune-TiO_2_NP recognition and interaction *in vitro*, confirming that primary immune cell cultures from *P. lividus* can be an optional model for swift and efficient immune-toxicological investigations.

## Introduction

Titanium dioxide nanoparticles (TiO_2_NPs) are very appealing nanomaterials for a variety of emerging applications. Unique chemical and physical features combined with limited toxicity, inertness, and biocompatibility make these nanoparticles one of the most highly employed nanomaterials for water purification, soil remediation, medical products, skin-care products, sensors, electronics, and many others (1). The same nanoparticles may also have distinctive bioactive properties promoting or preventing human and environmental health. The growing use of TiO_2_NPs stimulated an outbreak of competing research (from molecules to ecosystems) aimed at delineating the safe limits of TiO_2_NPs use in occupational or environmental exposures. This emphasizes the great level of interest concerning the biosafety of TiO_2_NPs. However, a complete and comprehensive understanding of the biosafety of TiO_2_NPs has not yet been achieved, due to large gaps of information in risk management, disposal, knowledge on the biomedical potentiality, interaction with many different biological factors (molecules) in environments and microenvironments, and due to ethical issues. Nanomaterials interact with living organisms through biological fluids (including blood, lung fluids, and bile fluids depending on the route of exposure), and act as a scaffold for biomolecules (2). The 3R principles of reduce, refine, and replace (3) have evolved into essential aspects for planning scientific trials that use animal models. New and more feasible approaches have resulted in the development of the novel *in vitro–ex vivo* methods to limit the use of living organisms (4). Based on this aim, we use primary cell cultures that accurately represent the biological tissue microenvironment in which cells reside, because cell–cell signaling remains preserved (5). Thus, primary cultures have become a much more appropriate tool for biotechnological applications and nano-safety/nano-toxicity investigations.

The new frontier in immuno-nano research has highlighted the sea urchin *in vitro-ex vivo* model in the practical and vigilant application of the 3R principle. This model has been launched recently by the European Partnership for Alternative Approaches to Animal Testing and Registration, Evaluation, Authorization, and Restriction of Chemical Substances regulation to enhance safety through the proper recognition of emerging chemicals/materials. Immunity is a purveyor of the internal equilibrium of organisms, and a century of research has uncovered the conserved means and mechanisms by which the immune system recognizes danger and regulates its own activity (6). Sea urchin genome sequence highlighted strong similarities between sea urchin and mammalian immunity, and is an exceptional example of sensing capacity and immune system complexity in an invertebrate (7). In many cases, sea urchin immune cells are the sole source of functional biomolecules that are secreted into the coelomic fluid (CF) (the echinoid body fluid that shares some common functions with those of higher animal blood) to maintain the proper internal equilibrium and intercellular crosstalk (8), and act as highly robust *in vitro* (4). *In vivo* studies on sea urchin immune cells demonstrate that immune cells from Mediterranean Sea urchin, *Paracentrotus lividus*, show selective changes in specific pathways when exposed to a range of nanoparticles (9, 10), similar to what is known in humans. For example, authors support the evidence that *P. lividus* phagocytes interacting with TiO_2_NPs elicit a receptor-mediated phagocytic mechanism involving the TLR/p38 MAPK signaling pathway, but do not activate an inflammatory response or the Hsc70-dependent stress response (10).

Growing evidence suggests that the interaction between nanoparticles and native or non-native proteins (*in vivo*/*in vitro)* forms a dynamic biomolecule coating or “corona,” representing bio-nano interface that is perceived by cells (2). This bio-nano interaction can be affected by many factors, especially in the marine environment and the internal body fluids of marine invertebrates (11).

However, there is little current knowledge on the secreted functional biomolecules involved in the immune nanoscale interactions with TiO_2_NPs and subsequent intracellular signal transduction, cellular trafficking and fate, particularly with regards to marine invertebrates.

Here, we characterize the behavior of TiO_2_NPs in a complex physiological medium, with a focus on the identification of sea urchin extracellular proteins that form the protein corona on the particle surface. We demonstrate that TiO_2_NPs acquire a “biological identity” quickly, and that the main constituents shaping sea urchin cell-conditioned TiO_2_NP protein corona are adhesion proteins (*Pl*-toposome, *Pl*-galectin-8, *Pl*-nectin) and other ligands that mediate phagocytosis (actin, tubulin). These results provide new insights into the sea urchin extracellular signaling implicated in immune-TiO_2_NP recognition and interaction *in vitro*, suggesting intriguing ideas about the use of sea urchin immune cells as a powerful tool for fast nano-safety/nano-toxicity immune investigations *in vitro*.

## Materials and Methods

### TiO_2_NP Source

Aeroxide TiO_2_ P25 nanoparticles (primary particle size ranging from 10 to 65 nm, irregular and semi-spherical shape; mesoporous NPs, anatase, and rutile 4:1) were purchased from Evonik Degussa (Essen, Germany) and characterized previously in several aqueous solutions (from freshwater to seawater) by transmission electron microscopy (TEM), Brunauer, Emmett and Teller method, and dynamic light scattering (DLS), as described by Brunelli et al. (12) and Pinsino et al. (10). TiO_2_NP stock suspension (100 μg mL^−1^) was prepared in ultrapure water (18.2 MΩ cm^−1^) (Purelab Option-Q System, UK), vortexed for 5 min and sterilized under UV light prior to use.

### Animals, Cell Cultures, and Sample Collection

Specimens of the sea urchin *P. lividus* were collected along the unpolluted coast of Sicily (Marine Protected Area, Italy) and were acclimatized and maintained under controlled conditions of temperature (16 ± 2°C), pH (8.1 ± 0.1), salinity (38–39%), and density (1.028–1.030 g/cm^3^). Animal handling, immune cell harvesting, and primary cell culture were performed as described by Pinsino and Alijagic (4). CF was collected diluted 1:1 in coelomocytes culture medium (CCM), and coelomocytes (immune cells) were counted and adjusted to 1.5 × 10^6^ cells/mL and seeded into each well of a 25-well plate (Thermo Fisher Scientific, UK). A TiO_2_NP suspension was immediately added to the immune cell cultures (1 μg mL^−1^ final concentration) and incubated at 16 ± 2°C for 24 h. At 3 and 24-h, medium was partially aspirated and cells were pelleted by centrifugation (9,000 × g, 10 min, 4°C) to purify cell-free CF plus CCM containing TiO_2_NPs, which was centrifuged a second time as described below. The adherent cells over the bottom of the culture well were gently dislodged with a soft scraper, aspirated into the remaining CCM, centrifuged (9,000 × g, 10 min, 4°C), and pellets were stored in aliquots at −20 and −80°C for future use.

### Quantitative *in vitro*-*ex vivo* Cell-Based Assays

All plate-based assays were performed in a 96-well white, opaque-walled plate (Thermo Fisher Scientific, UK) in a final volume of 100 μL. Immune cells were plated at a density of 1 × 10^5^ cells/well. TiO_2_NP exposure started upon cell plating by the gentle drop-by-drop introduction of the particles into the medium (0.1, 1, 10, and 100 μg mL^−1^ final concentration), and incubated in the dark at 16 ± 2°C. Immune cell samples were obtained from at least five sea urchins. After incubation with the TiO_2_NPs, cell viability and cytotoxicity were evaluated by RealTime-Glo MT Cell Viability Assay (Promega, USA), and the non-lytic CellTox™ Green Cytotoxicity Assay (Promega, USA), respectively, according to the manufacturer's instructions, with minor changes (4). MT Cell Viability Substrate plus NanoLuc Enzyme, and/or CellTox Green Dye (equilibrated to 16 ± 2°C) were added to the wells 21 h after exposure, and 3 h before the first real-time cell viability and cytotoxicity measurements (from 24 to 72 h of exposure to TiO_2_NPs). Luminescence and fluorescence were measured using GloMax Discover high-performance Microplate Reader (Promega, USA).

After 24 h of cellular exposure to particles, caspase 3/7 activity was evaluated by Caspase-Glo 3/7 Assay (Promega, USA). Hydrogen Peroxide (H_2_O_2_) levels were evaluated by ROS-Glo H_2_O_2_ Assay, according to the manufacturer's instructions, with minor changes. Plates were gently shaken for 30 min at 16 ± 2°C prior to the measurement.

### Nanoparticles Conditioning and Protein Corona Profiling

TiO_2_NPs were incubated in cell-free and/or cell-conditioned media in the dark at 16 ± 2°C in a plate shaker (bioSan, Latvia) for a maximum length of 24-h. TiO_2_NP incubation in cell-free medium (cell-free CF plus CCM) was prepared in TreffLab microcentrifuge tubes, whereas cell-conditioned medium was performed in a 25-well plate as described above (see Animals, cell cultures and sample collection section). After incubation, TiO_2_NPs with the associated proteins were recovered by centrifugation (21,130 × g, 20 min, 4°C) followed by three vigorous wash steps in ASW. The pellets of TiO_2_NPs with protein coronas were re-suspended with vigorous agitation in 10 μL of 2 × Laemmli sample buffer (Bio-Rad, USA) supplemented with β-mercaptoethanol, according to the manufacturer's instructions. Samples were boiled for 5 min to strip protein corona, and the TiO_2_NPs were removed by centrifugation (21,130 × g, 20 min, 4°C) following the method reported by Monopoli et al. (13) with slight modifications, as described. Eluted protein coronas were loaded into 4–20% Mini-PROTEAN TGX precast polyacrylamide gel (Bio-Rad, USA). Samples of media that were not incubated with TiO_2_NPs were included. Gels were run at a voltage of 150 V for 5 min and 100 V for 80 min. Gels were stained with Coomassie Brilliant Blue R250 to identify protein bands. All experiments were conducted at least in triplicate to ensure reproducibility.

To obtain the two-dimensional gel electrophoresis (2DE) map of the TiO_2_NP protein corona, eluted proteins recovered from 30 mL of cell-free CF plus CCM (1 μg mL^−1^ final concentration, 24-h of incubation with TiO_2_NPs) were incubated in a denaturing buffer [7 M urea, 2 M thiourea, 4% w/v CHAPS, 1% v/v Triton X-100, 40 mM Tris-HCl pH 8.8, 10 mM DTT (Sigma Aldrich), with 1.2% DeStreak (GE Healthcare), 0.2% v/v Bio-Lyte pH 3–10 (BioRad), 1% v/v Protease Inhibitor Mix (GE Healthcare), and 1 mM Na_3_VO_4_]. Samples were processed with ReadyPrep™ 2-D Cleanup Kit and ReadyPrep Reduction Alkylation Kit (BioRad), quantified by 2-D Quant kit (GE Healthcare), focused on 18 cm, pH 3–10 non-linear ReadyStrip™ IPG Strips (BioRad), and separated on 9–16% SDS-PAGE gel. Protein spots were detected by silver staining.

### Extracellular Proteins, Protein Corona, and Immunoblotting Analyses

After incubation with the TiO_2_NPs, culture medium was analyzed for protein content as follows. Samples were concentrated by membrane ultrafiltration with 5 kDa cut-off Vivaspin-500 concentrators (Sartorius, UK). The protein content of each sample were quantified by the Bradford method with the BioRad assay kit (Hercules, CA, USA). Prior to SDS-PAGE, extracellular proteins (secretomes) were mixed with acetone (1:1) for overnight precipitation at −20°C. Acetone-precipitated extracellular proteins were suspended in SDS sample buffer supplemented by the β-mercaptoethanol, boiled for 5 min and separated on 4–20% Mini-PROTEAN TGX precast polyacrylamide gels (BioRad, USA), and transferred to nitrocellulose membrane (Amersham, UK) according to standard procedures. Non-specific binding sites were blocked with Odyssey blocking buffer (LI-COR Biosciences, USA) for 1 h at room temperature (RT). After blocking, membranes were incubated with either one of the following primary antibodies in Odyssey blocking buffer with 0.1% Tween20: (i) anti-*Pl*-toposome (BEVIB12b8) 1:200 dilution; (ii) anti-*Pl*-nectin 1:200 dilution; (iii) anti-*Pl*-galectin-8 1:800 dilution; (iv) anti-Hsp70 (SIGMA, Cat N. H-5147) 1:1,000 dilution; (v) anti-P-p38 MAP Kinase (Tr180/Tyr182) (Cell Signaling, 9211) 1:250 dilution; (vi) anti-β-actin (SIGMA, A5441) 1:500 dilution; anti-α-tubulin (SIGMA, T5168) 1:2,000 dilution. Following rinsing, membranes were incubated with a fluorescein-labeled secondary anti-mouse and/or anti-rabbit antibody (LI-COR Biosciences) and visualized with Odyssey Infrared Imager (LI-COR Biosciences). Immunoblotting for TiO_2_NPs protein coronas were performed following the procedures described in this section for secretomes.

### Electron Microscopy Analysis

#### Scanning Electron Microscopy

Control and TiO_2_NPs-exposed immune cells (1 μg mL^−1^ final concentration, 24 h of exposure) that adhered to 12 mm circular glass coverslips (Menzel Gläser, Germany) were fixed with a 1:1 mixture of 6% glutaraldehyde in 100 mM cacodylate buffer and ASW (pH 8–8.5) for 1 h at RT. Coverslips were washed three times with cacodylate buffer, dehydrated through an ethanol series (25, 50, 75, 90, 96, and 100%), and dried in a K850 Critical Point Dryer (Quorum Technologies Ltd, Ringmer, UK). The dried coverslips were sputter coated with 3 nm platinum using high-resolution Turbo-Pumped Sputter Coater Q150T (Quorum Technologies Ltd, Ringmer, UK). The samples were examined in FEI Nova NanoSEM scanning electron microscope (FEI, Czech Republic) at 5 kV using CBS and TLD detectors.

#### Transmission Electron Microscopy

Immune cells were incubated in a 15 mL tube (Thermo Scientific, USA) (1 μg mL^−1^ final concentration for 24 h) with gentle agitation, followed by fixing as described above. After intensive washes, cells were post-fixed overnight at 4°C with 1% osmium tetroxide in cacodylate buffer. Samples were washed three times in cacodylate buffer at 4°C and double distilled H_2_O, warmed to the RT and embedded into 4% low-melt agarose. Solidified agarose was cut into 1 × 1 mm cubes, dehydrated in ethanol series (25, 50, 75, 90, 96, and 100%), and embedded into the epoxy resin (EMBed-812 Embedding kit; Electron Microscopy Sciences). Ultrathin sections were stained with uranyl acetate and lead citrate, and examined with a FEI Morgagni 268(D) electron microscope (FEI, Czech Republic).

### Statistics

Statistical software GraphPad Prism Software 6.01 (USA) was used for data analysis. Statistical differences were estimated by one-way or two-way ANOVA (followed by the multiple comparison tests) and compared to the appropriate control. The level of significance was set at *p* ≤ 0.05. Data were presented as mean ± SE. Gel scans were processed by ImageJ software (NIH, USA).

## Results and Discussion

### Cell-Conditioned Protein Corona on TiO_2_NPs and Extracellular Signaling Include Sea Urchin Adhesion Proteins and Other Ligands That Mediate Phagocytosis

The extracellular environment is highly dynamic due to the process of “cell-conditioning,” in which cells deplete and secrete biomolecules (14). When engineered particles encounter an extracellular environment, they are coated by a complex biological fluid composed of proteins and other biomacromolecules, forming a protein corona, and providing a “biological identity” of nanosized materials (15). The biological identity of the NPs depends on the protein repertoire that they encounter plus their composition and structural properties, which are crucial for NP interactions with living cells. Because we found that cell-free medium contains an average protein concentration of about 7.093 ± 0.565 μg mL^−1^ (mean ± SE), we postulated that incubation of TiO_2_NPs within this medium would result in the adsorption of proteins onto the particle surface. To support this notion, we investigated if, when, and which of the secreted proteins may be involved in the immune nanoscale interaction with TiO_2_NPs.

### Sea Urchin Protein Corona Profiling in a Cell-Free Biological Medium

**Figure 1:**
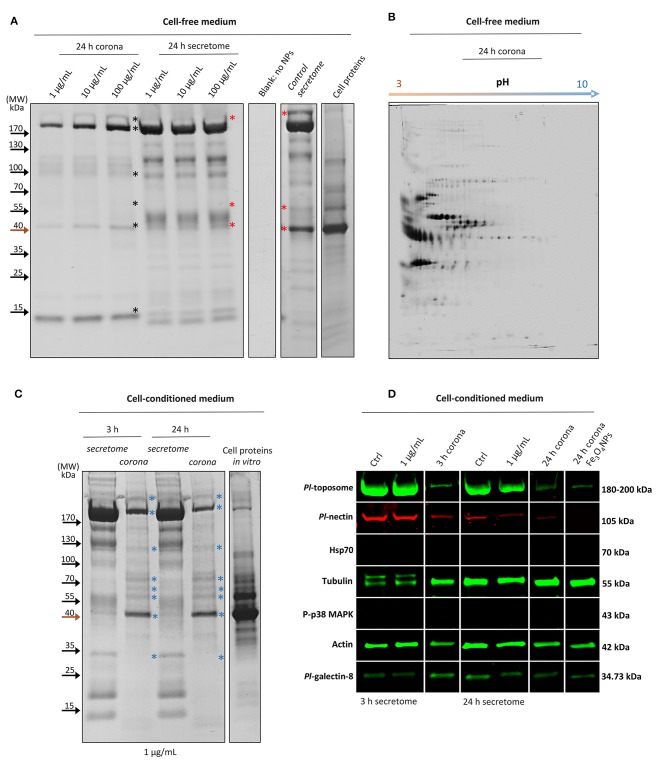
The interaction between Sea urchin secretome and TiO_2_NPs, extracellular signaling and protein corona identification. **(A)** One-dimensional profiling of the formed non-cell-conditioned protein corona at 1, 10, and 100 μg mL^−1^ TiO_2_NPs after 24 h of incubation. **(B)** Two-dimensional profiling of the TiO_2_NP protein corona after 24 h of incubation in the non-cell-conditioned medium (Cell free CF plus CCM). **(C)** One-dimensional profiling of the protein corona formed *in situ (in vitro)* at 3 and 24 h. **(D)** Representative immunoblotting results show limited influence of the TiO_2_NPs on sea urchin extracellular signaling and reveal the main constituents shaping the cell-conditioned protein corona (*Pl*-toposome, *Pl*-galectin-8, *Pl*-nectin, actin, tubulin). ^*^, see text.

To profile the sea urchin *P. lividus* corona proteins that have high affinity for the TiO_2_NPs, varying concentrations (1, 10, and 100 μg mL^−1^) of TiO_2_NPs were incubated with cell-free CF collected from donors diluted in the anticoagulant solution (CCM) containing EGTA (a chelating agent with a high affinity for Ca^2+^ that inhibits immune cell aggregation mediated by adhesion proteins) (4), followed by immediate centrifugation to pellet the particles. Proteins adsorbed onto the particle surface by 24 h were eluted, separated by reducing 1D SDS-PAGE, and labeled with Coomassie Brilliant Blue R250. Proteins adsorbed onto and extracted from the nanoparticle surface (first three lanes, namely 24 h corona) were compared to the total extracellular proteins (second three lanes, namely 24 h secretome) (Figure 1A). The process of corona formation is determined by the competition among proteins in the medium based on the affinity of each protein to the particle surface. A common feature in all protein corona samples was a selective enrichment of proteins of > 170 and about 100, 40, and 15 kDa (Figure 1A, black asterisks). There were other proteins adsorbed onto the particle surface, although to a much lesser extent, which was not easily visualized by 1D SDS-PAGE. We note that with increasing concentrations of particles, there were increased quantitative (but not qualitative) enrichments of the protein corona. Notably, a few proteins present in the corona showed a correlated reduction in TiO_2_NP-exposed secretomes compared to the control secretome in which the cell-free medium was not exposed to particles. Specifically, proteins ≥ 170, 55, 40 kDa showed strong bands in the control secretome but not in the TiO_2_NP-exposed secretomes (Figure 1A, red asterisks).

The analysis of interacting sea urchin extracellular proteins by 2DE permitted discrimination of the TiO_2_NP protein corona based on the pH of the proteins eluted from the particles. The 2DE map of the proteins from TiO_2_NPs (1 μg mL^−1^ final concentration) after incubation for 24 h in a cell-free medium showed that the majority of the proteins were negatively charged and distributed between the pH 3 and 6 (Figure 1B). Similarly, the TiO_2_NPs were also negatively charged between pH 7 and 8 (16) (this study: pH 7.4). The mechanism by which proteins are known to bind particle surfaces are principally hydrophobic/electrostatic interactions and hydrogen bonds, which are weak interactions. Here, both the TiO_2_NPs and the bound proteins were negatively charged, and therefore were not likely to form complexes based only on electrostatic interactions. Accordingly, the highly abundant proteins in the protein corona may be dependent on protein abundance, non-electrostatic affinity, solubility, and their degree of unfolding (17). For example, related studies on sea urchin extracellular protein/nanoparticle interaction that employed other engineered metal nanoparticles (e.g., iron oxide nanoparticles) resulted in a different protein corona profile compared to those of TiO_2_NPs (although there were some exceptions) (Supplementary Figure 1). This result supported the idea that TiO_2_NPs acquire a biological identity that may be based on selective affinity for a few proteins and a non-specific affinity for others. Notably, Fe_3_O_4_NPs are similar to TiO_2_NPs with regard to surface area, charge, and primary size, but have different crystalline structure and stability. Fe_3_O_4_NPs are well-known to have harmful effects on sea urchin immune cell biological functions *in vivo* (9).

It has been reported that proteins do not distribute randomly on particle surfaces, but form clusters in association with the first proteins that bind, which in turn attracts other proteins and continues until surface is filled (18). Therefore, mechanisms such as molecular relaxation and spreading depending on the time that proteins remain on the surface, can be a determining factor in making permanent attachment of the proteins.

Macromolecules of high molecular weight and/or negative charge favor steric or electrostatic colloidal TiO_2_NP stabilization (19). As is well-known, some ions of opposite charges adsorb onto NP surfaces, forming a Stern layer, while the high content in biomacromolecules—lipids, sugars, nucleic acids, and particularly proteins—alters the stability of NPs and changes surface chemical characteristics (20, 21). Moreover, divalent ions, such as Ca^2+^ and Mg^2+^, can act as an effective bridge to adsorb negatively charged proteins onto the negatively charged surface of TiO_2_NPs (22). It is therefore worth noting that CCM does not contain any organic molecules and is composed of divalent ions and a chelating agent with a high affinity for Ca^2+^ (4), whereas cell-free CF media contain functional biomacromolecules (8).

On the one hand, the high salt concentration of CCM tends to screen (reduce) the electrostatic repulsions between nearby nanoparticles, favoring homoaggregation (particle-particle complexes). On the other hand, the abundance of organic components suspended in cell-free CF media are likely to favor heteroaggregation (particle-protein complexes) (19).

### Sea Urchin Protein Corona Profiling in a Cell-Conditioned Medium

To investigate the sea urchin cell-conditioned protein corona formed upon contact with the TiO_2_NPs *in situ (in vitro*), TiO_2_NPs (1 μg mL^−1^) were incubated for 3 and 24 h with both not-induced and induced cell secreted-proteins (proteins that were putatively secreted *ex novo* during cell incubation) (Figure 1C). At this concentration of particles, sea urchin immune cells did not show any harmful effects on biological functions (see the section on protein corona architecture intercedes TiO_2_NPs association and clearance by immune cells). After 3 and 24 h, cell-conditioned TiO_2_NP protein corona profiles showed the same band pattern without any apparent rearrangement of proteins on the surface, perhaps because the most abundant proteins that bound the particle surface also have had the highest binding affinity (the Vroman effect) (23). The coronas appeared much more enriched in proteins than those obtained from cell-free biological medium at the same particle concentration (1 μg mL^−1^) (cell conditioning) (Figure 1C, bands indicated with blue asterisks; compare to bands with black asterisks in Figure 1A).

These findings were consistent with the notion that immune cells secrete immunologically active proteins that interact with particles (24) that contribute to the generation of the protein corona. This result is in agreement with the activities of immune cells from the earthworm *Eisenia fetida* exposed to silver nanoparticles (25). Notably, by pre-incubating the TiO_2_NPs for 6 h with bovine serum albumin (BSA) (10 and 100 μg mL^−1^) prior to immune cell exposure *in vitro* or by adding BSA (10 or 100 μg mL^−1^) and TiO_2_NPs into the immune cell culture simultaneously, caused changes in the TiO_2_NP protein corona profile (Supplementary Figure 2). In agreement with recent reports, BSA has high affinity for TiO_2_NP surfaces that act to reduce the capability of other proteins to be adsorbed onto the particle surface thereby altering cell culture behavior (Supplementary Figure 3). The protein/nanoparticle interaction ranges from opsonisation by immune or non-immune proteins for uptake by phagocytic cells, and determines the subsequent immunological cascade that can either stimulate or mitigate the immune response (26).

### Highlighting the Biological Identity of the TiO_2_NP-Protein Complexes in the Sea Urchin Immune Cells

The biological identity of a nanoparticle changes over time in the extracellular environment because of cell conditioning of the medium. To obtain a broad insight on the biological identity of the TiO_2_NPs *in vitro*, we identified a few key regulatory proteins that are putatively involved in the corona formation (Figure 1D).

Proteins for analysis were chosen by their approximate molecular weight that were comparable to bands identified by reducing SDS-PAGE, and based on proteins known to be involved in cellular adhesion (*Pl*-toposome, *Pl*-galectin-8, *Pl*-nectin), cytoskeleton organization (actin and tubulin), and immune/stress response (Hsp70, P-p38 MAPK). The toposome is essential for sea urchin cell adhesion and development, and is a modified iron-less calcium-binding transferrin of six identical polypeptides of 170 kDa (27). In the sea urchin *P. lividus*, both CF and immune cells contain the precursor of toposome (180–200 kDa) that can be secreted in large quantities into the CF upon injury (28–30). In agreement, the CF of immune cells maintained in culture for 3 and 24 h (both exposed to TiO_2_NPs and control) contained band of > 170 kDa consistent with the expected size of secreted toposome (Figure 1C). By immunoblotting, the toposome was detected as a large protein band with a molecular weight ranging from 180 and 200 kDa in both control and exposed samples (Figure 1D). The presence of a large amount of available toposome in the CF was not consistent with the minor band of ~200 kDa for extracted proteins in the corona (Figure 1D). The expectation was that the strong binding affinity with the particle surface would have resulted in a strong band in the eluted corona proteins. In contrast, two other well-known proteins involved in sea urchin cell adhesion (galectin-8 and nectin) appeared in much lower content, and their binding affinities to TiO_2_NPs were very similar (Figure 1D). Recently, studies have described the possibility that both galectin and nectin may take part in corona modelling (31, 32). It is known that galectin-8 functions in cell adhesion a substrate, which promotes their cytoskeletal organization, attachment, and spreading by triggering integrin-mediated signaling cascades (33). When galectin-8 is present in excess as a soluble ligand, it negatively regulates cell adhesion and inhibits cell-substrate adhesion (33). In contrast, nectin is involved in cell-cell adhesion and is essential for ontogenesis, regeneration, and tissue maintenance (34). Notably, the levels of these proteins in the sea urchin secretome exposed to TiO_2_NPs for 24 h was lower than that for the controls, perhaps because these proteins were sequestered on the particle surface. Both sea urchin toposome and nectin are glycoproteins (28, 35), while galectin-8 binds glycoproteins (36). Glycoproteins have good binding affinity to TiO_2_NP particle surface (37). Notably, glycoproteins may be very important in transport with good binding capacity for calcium (37), particularly given that calcium signaling has a key role in phagocytosis (38). The incorporation of cell-secreted proteins into the particle-protein complex may increase receptor affinity or may target additional receptors.

The main cytoskeleton proteins actin and tubulin were found to be strongly involved in the formation of the TiO_2_NP-corona complex both at 3 and 24 h (Figure 1D), suggesting a crucial role for these proteins in determining both TiO_2_NP stability and biological effect. As is well-known, actin and tubulin are present in the intracellular and extracellular space in a monomer/polymer dynamic exchange form (39, 40). Tubulin aids in maintaining cell shape and undergoes post-translational modifications (glutamylation and phosphorylation) to specify microtubule subpopulations for particular functions (41). In agreement with tubulin functions, after 3 hrs, anti-α-tubulin antibody recognized two bands rather than one, suggesting that the cultures contained unattached cells undergoing morphological rearrangement. Notably, only one of the bands was associated with the TiO_2_NP-corona complex. However, it could not be excluded that the two tubulin bands may be due to partial protein degradation resulting from the extraction procedure. Actin is a highly versatile and conserved protein that can be secreted upon immune challenge, and it is involved in mediating phagocytosis that lead to killing bacteria and inhibiting innate immune inflammatory cascades (42, 43). On the other hand, Hsp70 and phosphorylated p38 MAPK were not detectable in any extracellular environment. Hsp70 translocates to the plasma membrane after a stress challenge and is released into the extracellular environment in a membrane-associated form that activates immune cells (44). Our results indicated a general healthy state of sea urchin immune cells in culture with TiO_2_NP exposure, which was not perceived as a stress inducer that was confirmed because it did not stimulate the release of the Hsp70 into the extracellular environment. p38 MAPK is a member of intracellular kinases that serves as focal point for diverse extracellular stimuli (45), which was used here as negative control.

Sea urchin extracellular proteins of different function adhere with varying affinity to TiO_2_NPs, perhaps acting as the immunoregulatory biointerface between particles and immune cells. Notably, *Pl*-toposome, *Pl*-galectin-8, actin, and tubulin were also detected in the corona of Fe_3_O_4_NPs, whereas nectin was not detected (Figure 1D, last lane). The protein corona modifies the size and the surface composition of the NPs, giving them a new identity depending on a selective affinity for a few proteins and a non-specific affinity for others that determines the full physiological response.

### Protein Corona Architecture Intercedes TiO_2_NPs Association and Clearance by Immune Cells

*Paracentrotus lividus* CF includes three different immune cell populations; phagocytes, amoebocytes, and vibratile cells, that show a phagocytic cell prevalence (>80%) (46). Because CF may contain host-associated microbiota and other non-self particles, phagocytic activity is essential in the particle detection and clearance that maintains intracellular homeostasis (8). To focus on understanding the interaction between TiO_2_NPs and immune cells, sea urchin immune cell topography and related TiO_2_NP surface distribution was evaluated by SEM for samples after 24 h of exposure *in vitro* (1 μg mL^−1^ TiO_2_NPs final concentration).

**Figure 2:**
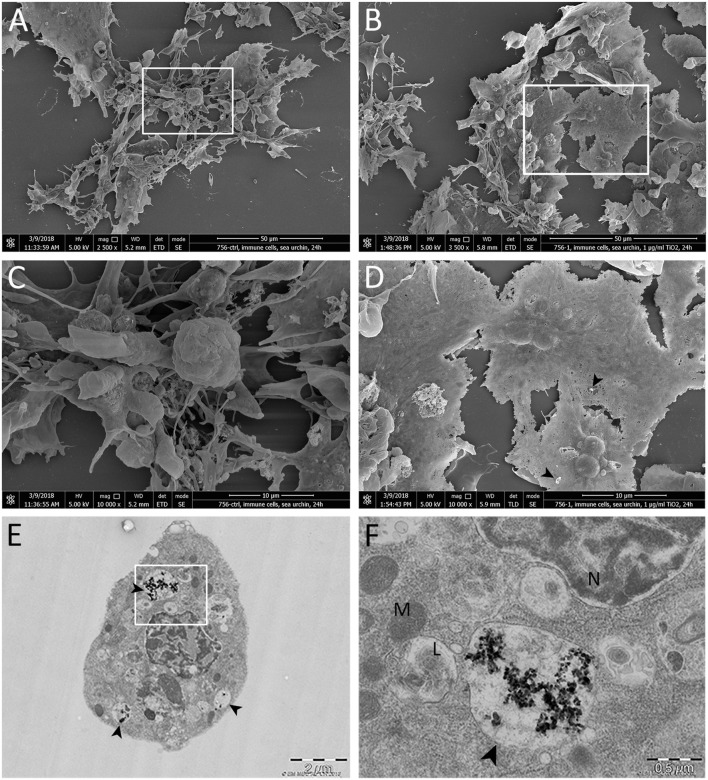
Scanning and transmission electron microscopy of the *in situ* (*in vitro*) interaction between TiO_2_NPs and sea urchin immune cells. **(A,C)** Control cells not exposed to TiO_2_NPs. **(B,D)** Immune cells exposed to 1 μg mL^−1^ TiO_2_NPs for 24 h. These figures illustrate interactions and selective binding of TiO_2_NPs on the immune cell (phagocyte) surface. Black arrowheads indicate small aggregate. **(E,F)** Sea urchin phagocyte after exposure to 1 μg mL^−1^ TiO_2_NPs for 24 h shows internalized TiO_2_NPs within a vesicular structure (black arrowhead) that is localized in the proximity with lysosomes (L). N, nucleus, M, mitochondria.

Phagocytic cells appeared strongly adherent and well spread to a greater extent than the control cells (compare Figures 2A,C to Figures 2B,D), revealing a tight adhesion that may have been mediated by the communication between cell surface receptors and protein ligands integrated into the biocorona or by any unbound surfaces of the NPs. No other cell types appeared to be involved in interactions with the TiO_2_NPs (not shown). A few small aggregates/agglomerates of the TiO_2_NPs protein complexes were adhered to the outer membrane surface of the sea urchin phagocytic cells (black arrowheads in Figures 2B,D). Notably, the TiO_2_NPs (1 μg mL^−1^) were internalized in well-organized cytoplasmic vesicles with ranging in size from 150 nm to 3 μm in diameter that were located toward the periphery of the cell near the plasma membrane (Figures 2E,F). This result was in agreement with our recent reports that TiO_2_NPs stimulate sea urchin immune cell phagocytic activity that involves the TLR/p38 MAPK signaling pathway *in vivo* (10). No disruption of the internal cellular membranes or cytoplasmic structures or organelles were noted. The TiO_2_NPs-protein complexes on the particle surface may function to enhance particle internalization by a broad spectrum of ligand bound receptors and cytoskeletal elements that are known to mediate internalization. These results suggest that cells take up these particles very promptly, safely and efficiently. We speculate that to maintain immune resilience toward the TiO_2_NPs, the TiO_2_NPs-protein complexes are recognized by the immune cells as “self,” thereby not eliciting any harmful immune effects of the particles.

**Figure 3:**
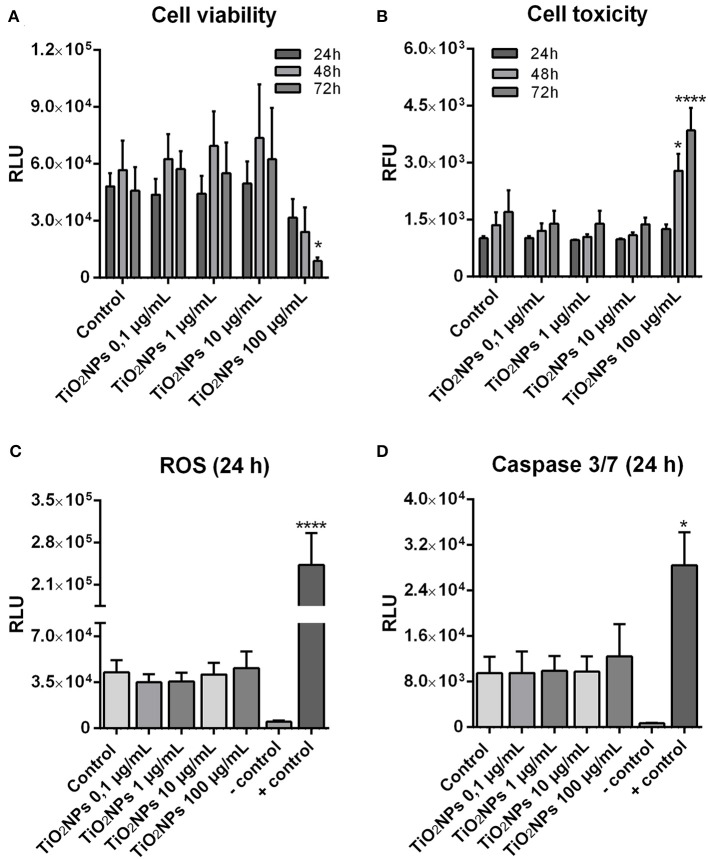
Impacts of TiO_2_NPs on the viability, toxicity, reactive oxygen species production and caspase activity on sea urchin immune cells. **(A)** Real-time viability assay over 72 h shows that only the highest dose of TiO_2_NPs (100 μg/mL) induces decrease in cell viability. **(B)** Cell toxicity shows an increase only in response to the highest TiO_2_NPs dose. **(C)** A Luciferase-based ROS assay does not indicate any significant increase in the ROS levels in response to TiO_2_NPs. Positive control (+ control): Hydrogen peroxide activates a strong increase in ROS production (24 h, 4 mM). **(D)** Caspase 3/7 activity shows that TiO_2_NPs (at all doses) does not induce an increase in the activity of these apoptotic enzymes. The positive controls (+ control) are cells exposed for 24 h to Iron oxide nanoparticles (100 μg/mL). Assays involved five biological replicates except for Caspase 3/7 assay, which had three replicates. Data are reported as the mean ± SE; stars (*) indicate significant differences among groups (*p < 0.05; ****p < 0.0001). RLU, Relative Luminescence Units; RFU, Relative Fluorescence Units. Negative control (- control) was medium plus particles without cells.

To support our speculation on the safety of the TiO_2_NPs, cell viability, cytotoxicity, caspase activity, and reactive oxygen species production were monitored in real-time measurement for cells exposed at increasing concentrations of the NPs (0.1, 1, 10, 100 μg mL^−1^) (Figure 3). The viability and cytotoxicity of exposed immune cells were similar to the controls during the 3 days of continuous monitoring, of which three measurement points (24, 48, 72 h) are shown. An exception was indicated for those cells exposed to the highest TiO_2_NP concentration (100 μg mL^−1^), which showed significantly decreased viability and significantly high toxicity (Figures 3A,B). For samples exposed to 0.1 to 10 μg mL^−1^ TiO_2_NPs, the RealTime-Glo MT cell viability assay indicated an increased trend (not statistically significant) in cell viability/metabolic activity compared to unexposed controls. Metabolites are largely controlled by extracellular signals, which direct the uptake, storage, and utilization of substrates (glucose, amino acids, nucleotide and fatty acids) (47). In turn, metabolites control the duration and intensity of innate or adaptive immune activation and memory cell formation. Studies elucidating metabolic profiles of immune cells exposed to TiO_2_NPs are necessary to clarify this fascinating result. We are continuing this analysis and our preliminary results suggest an increase of a few antioxidant metabolic pathways under exposure to TiO_2_NPs (1 μg mL^−1^ final concentration) (unpublished data).

ROS production and caspase 3 and 7 levels did not show a significant dose-dependent increase in cells exposed to TiO_2_NPs (Figures 3C,D). In sea urchins, modest physiological levels of intracellular ROS are required throughout their entire lifespan (48). Powerful antioxidant machinery maintains sea urchin genomic stability through the activation of DNA repair actions, enabling long life and bypassing the outcomes of ageing (8). Critically, our findings support the notion that *P. lividus* immune cells act according to a simultaneously biphasic strategy: resistance and tolerance. Under homeostatic conditions, constitutive immunity maintains physiological order within the organism. Whereas, under perturbations such as TiO_2_NP exposure, the immune system induces a short-term and energetically expensive response perhaps explaining increase in metabolic activity that may result in the long-term activation of survival based on promoting tolerance (49, 50).

## Conclusions

To the extent of our knowledge, this is the first study to describe an enriched cell conditioned TiO_2_NP protein corona profile from marine invertebrate immune cells in culture. The characterization of proteins bound to the NP surface is the first step toward understanding the true nature of NP-mediated biological effects. Living cells secrete extracellular molecules to retain cell cycle, nutrient uptake, cell signaling, and migration, and to cope with different foreign entities (51). These macromolecules are receiving much attention as they modify NPs, which affects their stability and fate that may stimulate or mitigate the immune response. Here, we demonstrated that several sea urchin extracellular proteins interact with TiO_2_NPs and bind swiftly to the particle surface. Recent studies describing NP-protein complexes formation on invertebrates highlighted the involvement of only single protein surrounding the NP surface (25, 52). In contrast, here we show that the main constituents shaping the TiO_2_NP protein corona from media conditioned by sea urchin immune cells are a subset of adhesion proteins and cytoskeletal proteins. This is in agreement with the most recent knowledge concerning a broad spectrum of proteins that form human NP-protein complexes (e.g., immunoglobulins, lipoproteins, complement components, acute-phase proteins, coagulation factors) (26). This finding is not surprising, given that the genome of *Strongylocentrotus purpuratus* sea urchin shows the close genetic relationship between sea urchins and humans (7), which further reinforces the relevance of this model organism as *a proxy* to humans. Protein corona formation on the surface of TiO_2_NPs modifies the physicochemical properties of the particles and changes their behavior in biological microenvironments, leading to aggregation and, in turn, the behavior of the interacting cells, leading to adherence, spreading, uptake capability, and increased metabolism. The TiO_2_NP-protein complex formation is mainly due to the intrinsic properties of extracellular proteins that form the corona (e.g., protein abundance, affinity, solubility). These proteins may have selective particle affinity (*Pl*-nectin) and/or non-selective affinity (e.g., *Pl*-toposome, *Pl*-galectin-8, actin and tubulin) for the TiO_2_NPs, as suggested by differences in protein complex formation on Fe_3_O_4_NPs and the BSA competition assay. Nectins are cell adhesion molecules that mediate the formation of cell adherence junctions. From an immunological point of view, nectins have become of particular interest due to their well-known involvement in mediating the immune responses of different immune cell types during interactions with several immune modulatory receptors expressed at the cell surface of immune cells (53). Our results provide evidence that the uptake mechanism for TiO_2_NP may be the outcome of receptor–ligand (e.g., nectin) interaction, even if a non-specific passive uptake mechanism cannot be excluded. Because nectin, toposome, galectin-8, actin, and tubulin first interact with the physical features of particles, these proteins are likely to have important roles in the recognition of the non-self material, both biological and non-biological by the sea urchin immune cells.

## Data Availability Statement

The datasets generated for this study are available on request to the corresponding author.

## Author Contributions

AP: conceptualization. AP and AA: methodology, data curation, and writing–original draft. AA: validation and formal analysis. AA and DC: cellular/biochemical investigation. AA, OB, and OK: SEM and TEM investigation. AP: writing-review, supervision, project administration, and funding acquisition.

### Conflict of Interest

The authors declare that the research was conducted in the absence of any commercial or financial relationships that could be construed as a potential conflict of interest.

## Supplementary Material

Supplementary Figure 1 2DE map of the proteins from Fe_3_O_4_NPs (1 μg mL^−1^ final concentration) after incubation for 24 h in a cell-free medium. The majority of the proteins are distributed between the pH 3 and 10 and are negatively charged, and show a different protein corona profile compared to TiO_2_NPs (although there were some exceptions). Results confirm that each particle is able to acquire a selective “biological identity” depending on the particle-protein affinity.

Supplementary Figure 2 TiO_2_NP protein corona competition assay. **(A)** One-dimensional profiling of the protein coronas formed on particles pre-incubated with BSA (10 and/or 100 μg mL^−1^) prior to immune cell exposure *in vitro* show the almost exclusive presence of the BSA (protein band of about 70 kDa). **(B)** One-dimensional profiling of the protein coronas formed on particle surface adding BSA (10 or 100 μg mL^−1^) and TiO_2_NPs (1 μg mL^−1^) into the immune cell culture simultaneously, show the presence of the BSA and the exclusion of the major sea urchin proteins. BSA has high affinity for TiO_2_NPs and reduce the capability of the sea urchin extracellular proteins to be adsorbed onto the particle surface (compare Figure 1C and Supplementary Figure 2). On the left: secretome samples; on the right: protein corona samples.

Supplementary Figure 3 Protein corona changes affect cellular behavior. **(A)** Sea urchin immune cells exposed to TiO_2_NPs for 24 h show well adherent phagocytes that spread their protrusions across the plate and maintain cell individuality. Amoebocytes morphology indicates the healthy state of these cells, as they do not show sign of evident degranulation and maintain an ovoid shape. **(B)** Immune cells exposed to BSA (10 μg mL^−1^) and TiO_2_NPs (1 μg mL^−1^) simultaneously, show a slightly reduced capability to maintain an uniform distribution, moderate clustering and ovoid amoebocytes. **(C)** Immune cells exposed to BSA (100 μg mL^−1^) and TiO_2_NPs (1 μg mL^−1^) simultaneously, show aggregation (cell-cell clustering) perhaps forming multinucleated syncytia, a reduced capability to form fine network fibers, and circular amoebocytes (>50%).
