# Supplementary figures and images for: Sea Urchin Extracellular Proteins Design a Complex Protein Corona on Titanium Dioxide Nanoparticle Surface Influencing Immune Cell Behavior
Source: Front Immunol. 2019 Sep 20;10:2261. doi: 10.3389/fimmu.2019.02261 (PMC6763604; doi:10.3389/fimmu.2019.02261)

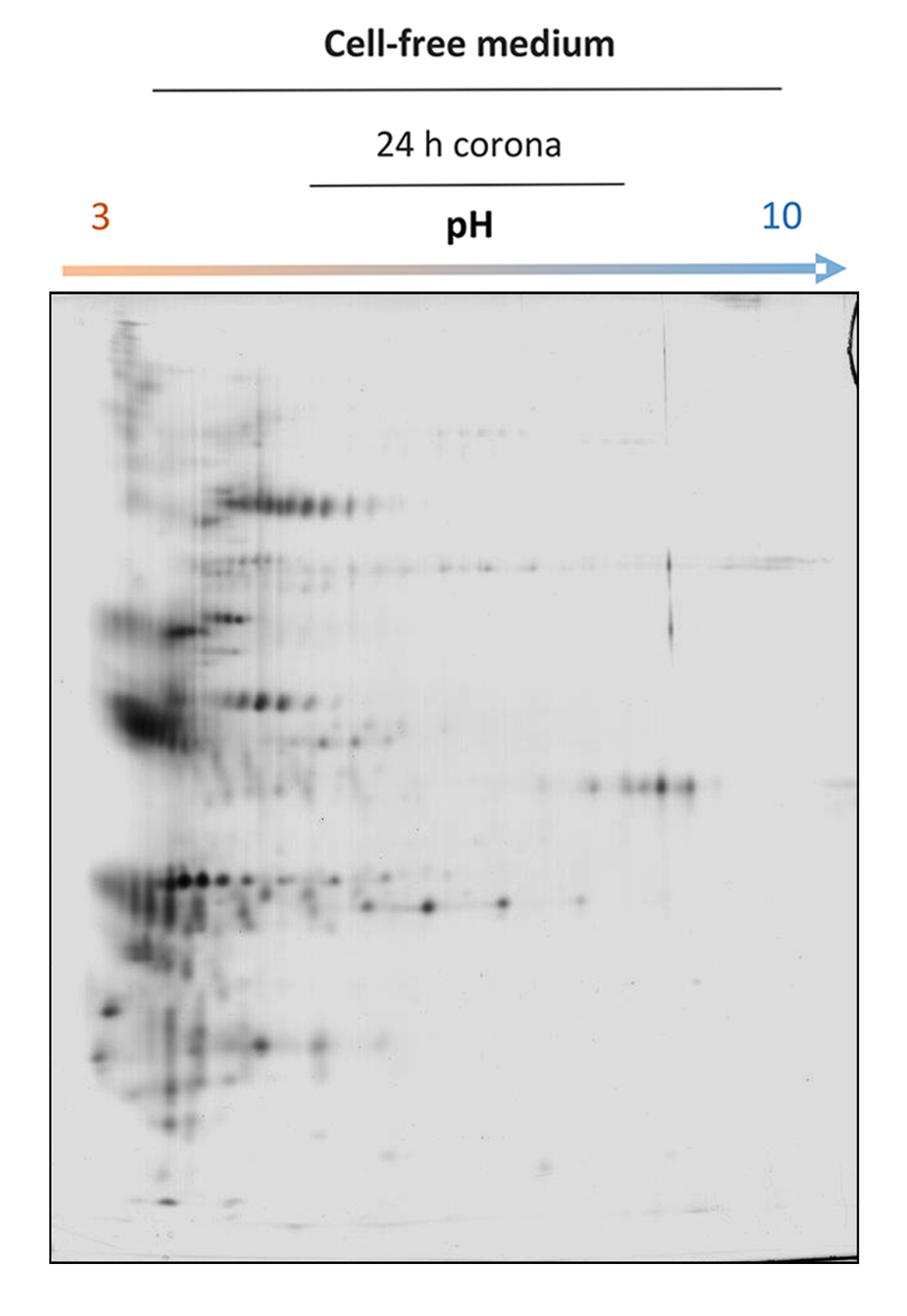

Supplement: Supplementary Figure 1 — 2DE map of the proteins from Fe3O4NPs (1 μg mL−1 final concentration) after incubation for 24 h in a cell-free medium. The majority of the proteins are distributed between the pH 3 and 10 and are negatively charged, and show a different protein corona profile compared to TiO2NPs (although there were some exceptions). Results confirm that each particle is able to acquire a selective “biological identity” depending on the particle-protein affinity. [file Image_1.TIF]

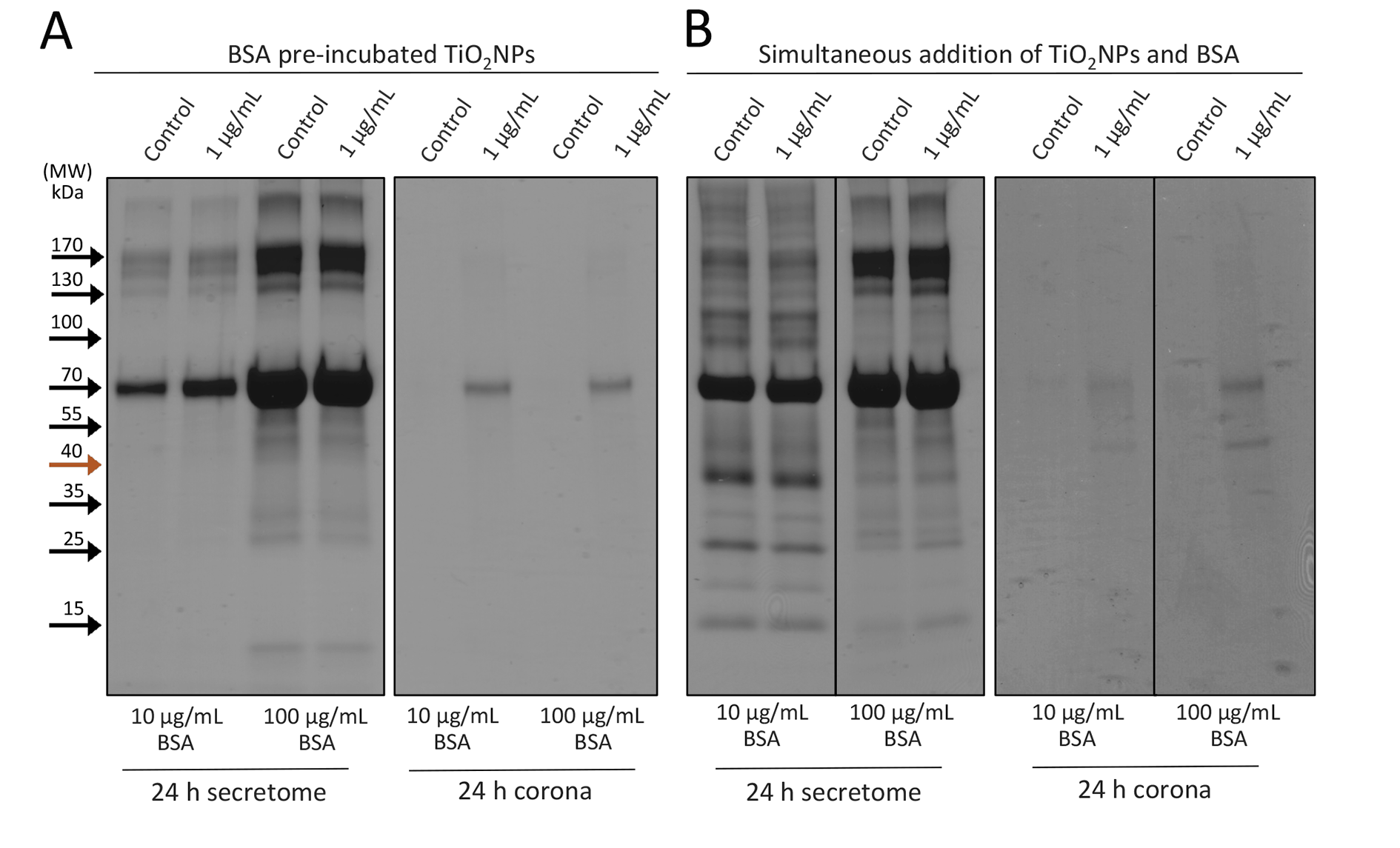

Supplement: Supplementary Figure 2 — TiO2NP protein corona competition assay. (A) One-dimensional profiling of the protein coronas formed on particles pre-incubated with BSA (10 and/or 100 μg mL−1) prior to immune cell exposure in vitro show the almost exclusive presence of the BSA (protein band of about 70 kDa). (B) One-dimensional profiling of the protein coronas formed on particle surface adding BSA (10 or 100 μg mL−1) and TiO2NPs (1 μg mL−1) into the immune cell culture simultaneously, show the presence of the BSA and the exclusion of the major sea urchin proteins. BSA has high affinity for TiO2NPs and reduce the capability of the sea urchin extracellular proteins to be adsorbed onto the particle surface (compare Figure 1C and Supplementary Figure 2). On the left: secretome samples; on the right: protein corona samples. [file Image_2.TIF]

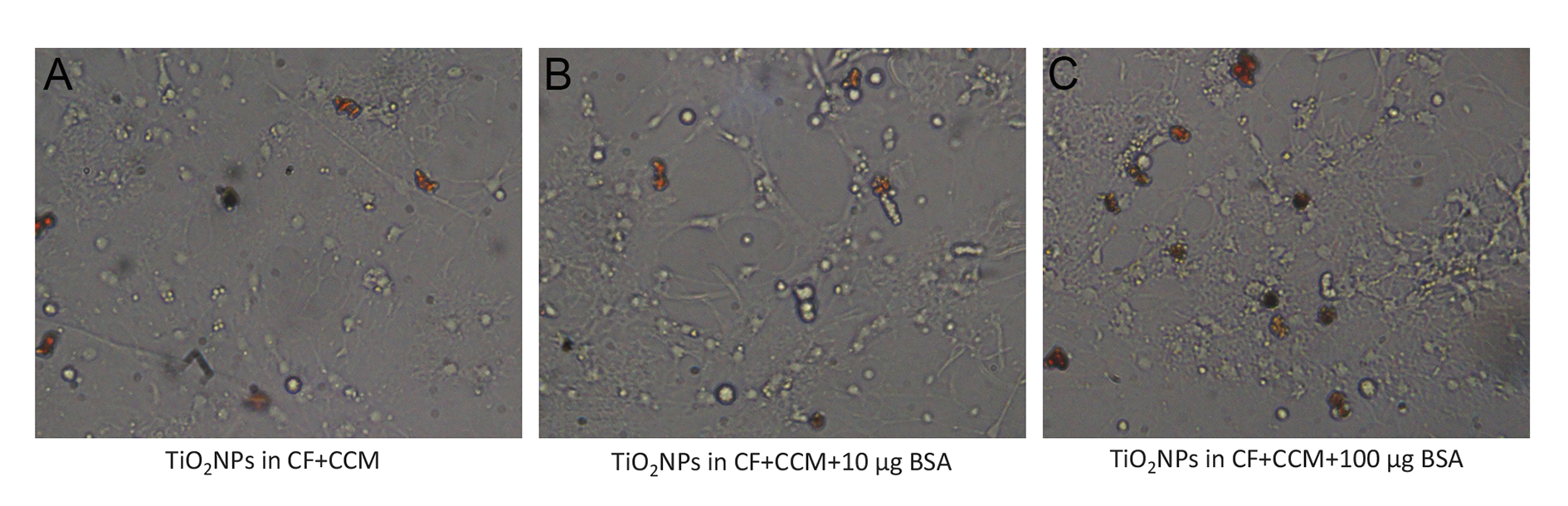

Supplement: Supplementary Figure 3 — Protein corona changes affect cellular behavior. (A) Sea urchin immune cells exposed to TiO2NPs for 24 h show well adherent phagocytes that spread their protrusions across the plate and maintain cell individuality. Amoebocytes morphology indicates the healthy state of these cells, as they do not show sign of evident degranulation and maintain an ovoid shape. (B) Immune cells exposed to BSA (10 μg mL−1) and TiO2NPs (1 μg mL−1) simultaneously, show a slightly reduced capability to maintain an uniform distribution, moderate clustering and ovoid amoebocytes. (C) Immune cells exposed to BSA (100 μg mL−1) and TiO2NPs (1 μg mL−1) simultaneously, show aggregation (cell-cell clustering) perhaps forming multinucleated syncytia, a reduced capability to form fine network fibers, and circular amoebocytes (>50%). [file Image_3.TIF]
